# The Efficacy of Corticosteroids, NSAIDs, and Colchicine in the Treatment of Pediatric Postoperative Pericardial Effusion

**DOI:** 10.1007/s00246-022-02820-4

**Published:** 2022-01-21

**Authors:** Nirmiti Somani, Hans Breur

**Affiliations:** Department of Pediatric Cardiology, University Medical Center Utrecht, Lundlaan 6, 3584 EA Utrecht, The Netherlands

**Keywords:** Postoperative pericardial effusion, Pediatric, Cardiac surgery, Colchicine, NSAIDs, Corticosteroids

## Abstract

The objective of this study is to investigate and compare the efficacy of corticosteroids, NSAIDs, and colchicine in treating postoperative pericardial effusion (PPE) following cardiac surgery in the pediatric setting, on the basis of available literature. To investigate and compare the efficacy of corticosteroids, NSAIDs, and colchicine in treating postoperative pericardial effusion (PPE) following cardiac surgery in the pediatric setting, on the basis of available literature. A systematic review was conducted by carrying out a database search in PubMed on April 20th, 2021. An English language filter was added, but no time restrictions were applied. Lack of pediatric literature prompted a broadening of the search to include adult literature. One pediatric and four adult studies were included, but the pediatric evidence was not found to be of satisfactory quality, and the findings of adult literature could not be readily generalized to the pediatric setting. No well-founded conclusions could be drawn regarding the efficacy of corticosteroids, NSAIDs, or colchicine in treating PPE, as a striking lack of evidence for their efficacy in the pediatric setting were revealed. A knowledge gap was found in the literature, indicating a need for good-quality randomized controlled trials to bridge this gap.

## Introduction

A common complication observed in patients following cardiac surgery is the development of pericardial effusion (PE) or the accumulation of excess fluid in the space around the heart. This can lead to life-threatening cardiac tamponade. Clinical signs of such postoperative pericardial effusion (PPE) include shortness of breath (dyspnea), malaise, discomfort or pain in the chest, low blood pressure, tachycardia, fever, and reduced urine output [1]. However, PPE can also present with non-specific symptoms or even asymptomatically [1]. The diagnosis of PPE can be carried out in a variety of ways, but the use of echocardiography and computed tomography has been reported in the literature [1–3].

While the precise pathogenesis of PPE remains to be elucidated, some theories have been proposed in the literature. The immune system is commonly implicated, for instance, with suggestions that an inflammatory mechanism is involved [2–4]. It has also been suggested that the development of PPE is the result of an autoimmune reaction, wherein the immune system produces antibodies against self-antigens that are exposed when the pericardium is damaged during surgery [3, 4]. In fact, this theory may explain why younger children, who do not yet have a completely developed immune system and older adults, whose immune systems show a decline in competency, tend to exhibit lower incidence rates of clinically relevant PE [3].

Many studies have been performed to ascertain the incidence of this complication, but a wide range of values can be found in the literature, ranging from estimates as low as 1.1% to those as high as 6.2%, subject to variations in study design, sample size, and other factors [2, 5]. Moreover, certain surgical procedures have been found to be associated more with this complication, than others; for instance, a study by Moh et al. found that patients undergoing coronary artery bypass grafting were more likely to develop pericardial effusion post-surgery than those who underwent valve replacements or other types of surgery [6].

A variety of factors have been suggested to influence the risk of developing PPE. Several studies in the literature have performed uni- and multivariate analyses to determine the factors that have a statistically significant impact on the likelihood of developing PPE [1, 3] or on the likelihood of requiring readmission to the hospital with PE [5]. The findings of these studies have been summarized in Table 1.

**Table 1:** Factors influencing the likelihood of developing or requiring readmission to the hospital with PPE in adults and in children [1, 3, 5]

Increased risk	Decreased risk
Adults	
Increased body surface area [1]	Prior cardiac surgery [1]
Thromboembolism in the lung [1]	
Kidney failure [1]	
Prolonged cardiopulmonary bypass time [1]	
Type of procedure: aortic root surgery [1]	
Children	
Higher age [3, 5]	Prior cardiac surgery [3]
Increased Continuous Positive Airway Pressure therapy (CPAP) duration [3]	Type of procedure: patent ductus arteriosus repair, ventricular septal defect closure, conduit, and electrophysiology surgical procedures [5]
Increased body surface area [3]	
Cardiopulmonary bypass [3]	
Use of inotropic agents [3]	
Down Syndrome [5]	
Type of procedure: cardiac transplant, systemic-pulmonary artery shunt, atrial septal defect closure (via surgery) [5]	

Given the prevalence of PE as a postoperative complication, one would expect a wide range of literature providing evidence for the effectiveness of the various methods of drug treatment reportedly being used. However, this does not seem to be the case. While many different approaches have been described for drug-based treatment of PPE in the literature, ranging from aspirin [2, 5, 6], non-steroidal anti-inflammatory drugs (NSAIDs) [2, 3, 5, 6], and corticosteroids [5, 6] to colchicine [2, 3, 5, 6], not many studies have compared these approaches to one another in an attempt to elucidate which one is most effective. This is especially true in the pediatric setting, wherein literature on the effectiveness of individual drug treatment approaches is scarce to begin with. Thus, this systematic review will investigate the following question: which method of drug treatment is most effective for treating PPE in children following cardiac surgery.

## Methods

A systematic literature search was performed using PubMed. The search terms included the MeSH terms ‘pericardial effusion,’ ‘postpericardiotomy syndrome,’ ‘postoperative care,’ ‘anti-inflammatory agents, non-steroidal,’ ‘colchicine,’ and ‘adrenal cortex hormones’ in various combinations with cardiac surgery, drug therapy, therapeutic use, and so on. The detailed search strategy can be found in Appendix 1.

The studies were selected on the basis of pre-determined criteria such that the participants must be human, the studies must be published in English, must have as their outcome, the size (width, or volume, assessed by means of an echocardiography) and/or clinical signs of postoperative pericardial effusion (PPE) following cardiac surgery (early or late onset), must investigate the influence of drug-based treatments (specifically, colchicine, corticosteroids, or NSAIDs) on the outcome, and must either be open access or accessible through the Utrecht University library. Initially, only pediatric literature was sought, but upon finding a striking scarcity of literature in this age group, the search was broadened to include adult literature, in order to attempt a generalization of the latter’s findings to the pediatric setting. No publication date restrictions were imposed.

## Results

**Fig. 1:**
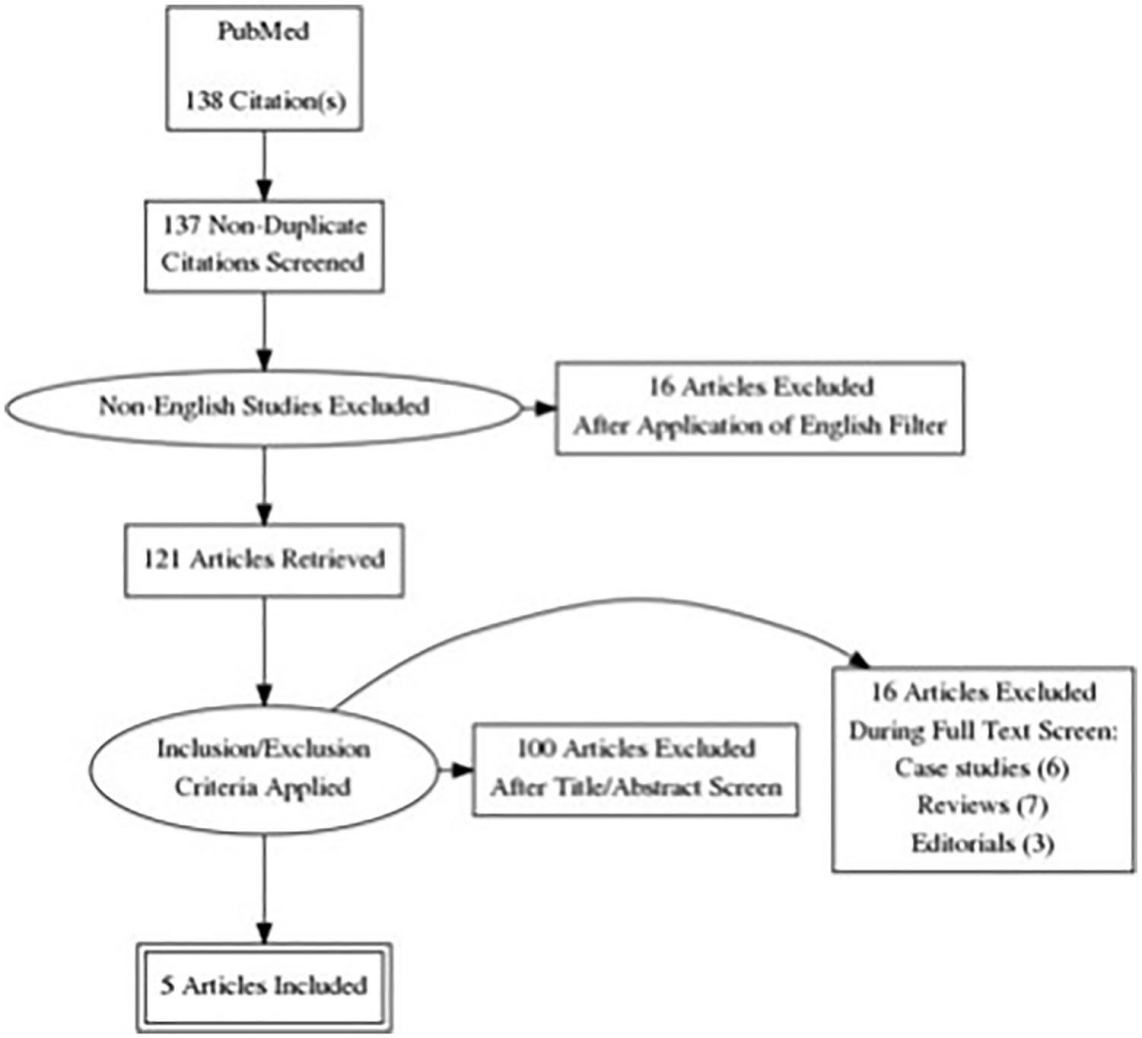
Study flow diagram detailing the step-by-step process undertaken during the literature search for this review. The syntax entered into PubMed (see Appendix 1. for details) yielded 138 articles or 137 non-duplicate articles. Of these, 16 were excluded after the English filter was applied, another 100 were excluded after the title/abstract screen, and finally another 16 articles were excluded after the full-text screen. Five articles were finally included in the review

Studies investigating the impact of prophylactic drug-based treatments for PPE (*n* = 26) or PE developed as a result of causes unrelated to cardiac surgery (such as neoplastic causes) (*n* = 6), having animals as subjects (*n* = 1), and published in a non-English language (*n* = 16) were excluded. Case studies (*n* = 6) and reviews (*n* = 7) were later excluded. The selection process of papers can be visualized in the study flow diagram in Fig. 1.

**Fig. 2:**
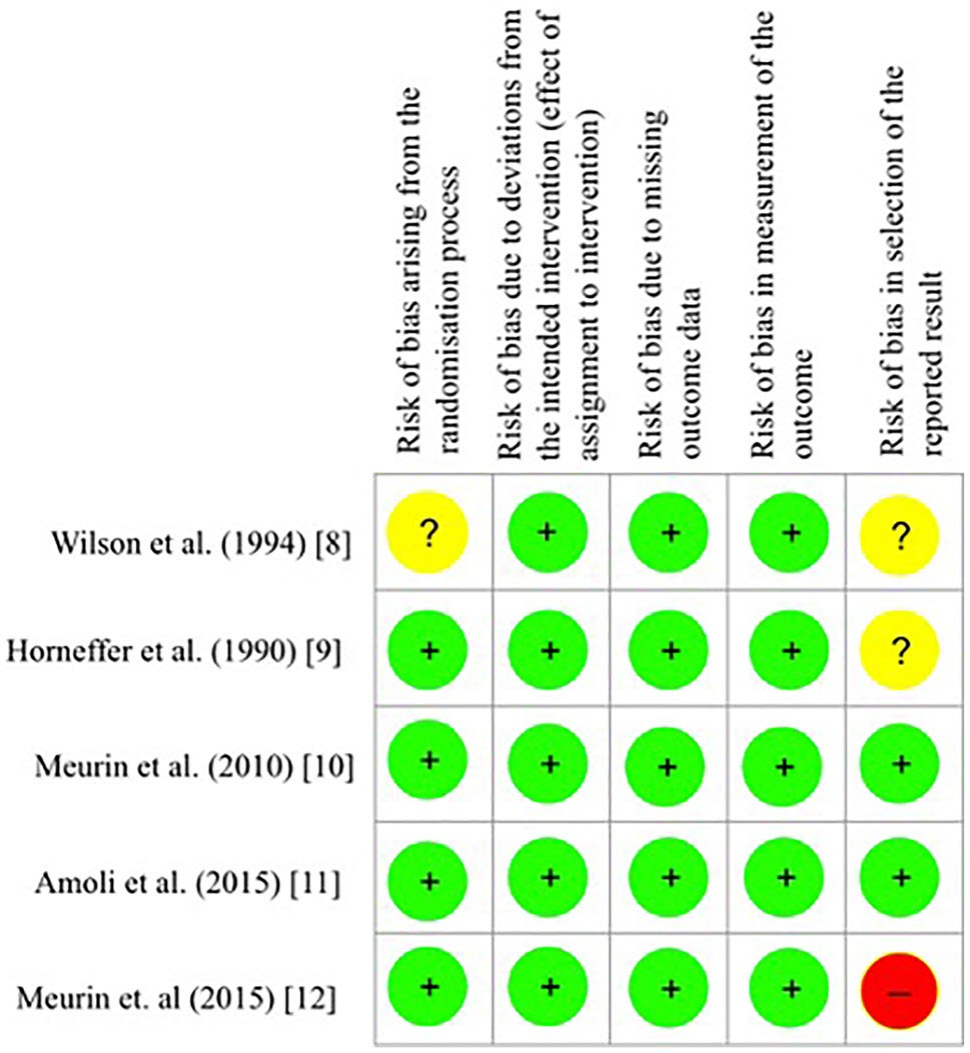
Summary of risk of bias assessment carried out using the Cochrane Risk of Bias 2.0 assessment tool [7]. “?” indicates some concerns, “+” indicates low risk of bias, and “−” indicates high risk of bias. See text for details

One study was selected in the pediatric setting [8] and four in the adult setting [9–12]. The eligible studies were assessed for quality of evidence using the Cochrane Risk of Bias assessment tool, version 2.0 [7]. The pediatric study was judged to raise some concerns regarding possible bias in the randomization procedure and in selection of the reported result. The adult studies were all judged to have low risk of bias, except for [9, 12], which were judged to raise some concerns or be at high risk (respectively) over bias in selection of the reported result. The domain-wise risk of bias assessment can be visualized in Fig. 2.

### Corticosteroids

A study by Wilson et al. [8] followed 290 children after they had undergone cardiac surgery. Of these, 21 were enrolled in the study (see Table 2 for inclusion criteria) and randomly assigned to the prednisone group (*n* = 12) or to the control group (*n* = 9). The former group was administered a prednisone suspension, while the latter group was given placebo. For the duration of the study, patients were not given any NSAIDs, including aspirin. Only simple analgesics like paracetamol were prescribed if required. Among other observations, the researchers studied the proportion of participants in each group who were in complete remission at 72 h and 1 week after the treatment had been started. The researchers defined remission as “the complete absence of all symptoms and signs of [PPS] for at least 24 h with static or decreasing effusions” [8, p. 63]. The researchers also measured the time until resolution of the patients’ effusions [8].

**Table 2:** Overview of literature on efficacy of corticosteroids in treating PPE in the pediatric setting [8]

Study	Participants	Type of study	Drug used	Dosage	Duration	Inclusion criteria	Definition of success	Results
Wilson et al. [8]	Children > 6 months of age (*n* = 21)	RCT	Prednisone, *n* = 12 (comparison with: placebo, *n* = 9)	2 mg/kg/day (reduced to 0 by day 14)	14 days	Diagnosis of PPS [when ≥ 2 criteria met: (1) fever > 37.8 °C; (2) characteristic clinical signs of pericarditis; (3) PE diagnosed with echocardiography, with or without diagnosis of pleural effusion] Procedures: repair of/operation–ASD, AVSD-PAB, L-TGA, Mustard, Mustard baffle leak, PA/VSD, tetralogy, truncus, VSD, ASD/PS, Rastelli (TGA/VSD/PS)	Primary outcome: Proportion of participants in each group showing complete remission at 72 h and at 1-week post-initiation of treatment and time to resolution of effusions	Significant differences found between prednisone and placebo groups (*p* = 0.03)

Findings from Pediatric Literature

*RCT* randomized controlled trial, *PPS* postpericardiotomy syndrome, *ASD* atrial septal defect, *AVSD* atrioventricular septal defect, *PAB* pulmonary artery banding, *L-TGA* corrected transposition of the great arteries, *PA* pulmonary atresia, *VSD* ventricular septal defect, *PS* pulmonary stenosis, *TGA* transposition of the great arteries

The main finding was that, while no significant differences were found at 72-h post-initiation of treatment, at 1 week, a larger proportion of the prednisone group was in remission. The difference between the groups at 1-week post-initiation of treatment was statistically significant (*p* = 0.03). It was also found that the prednisone group showed a trend toward faster resolution of effusions [8]. An overview of this study can be found in Table 2.

### NSAIDs

Horneffer et al. [9] followed 1019 patients after cardiac surgery, of which 149 were enrolled in the study (see Table 3 for inclusion criteria) and randomly assigned to one of three groups initially—an ibuprofen group, indomethacin group, or placebo group. Patients were not given any aspirin for the duration of the study (any aspirin prescribed prior to enrollment was discontinued, to be resumed only at the end of the 10 days of treatment). Only non-aspirin or acetaminophen-containing analgesics were administered if requested. However, at 48-h post-initiation of treatment, the researchers assessed the patients and found that treatment in a number of participants (*n* = 74) had clearly failed (defined by persistence of one or more of the symptoms used to make a diagnosis of PPS) and required intervention. At this point, the study drug code was broken and a preliminary analysis of the data was conducted, revealing that of the patients in whom treatment had failed, a majority belonged to the placebo group (*p* < 0.02). Next, the patients were randomized into one of two groups—the ibuprofen group or indomethacin group—for the remaining duration of the study. The study found that 90.7% of patients in the ibuprofen group, 87.5% of those in the indomethacin group, and 59.1% of those in the placebo group showed resolution of PPS symptoms. These differences were found to be statistically significant (*p* = 0.002) [9].

**Table 3:** Overview of literature on efficacy of NSAIDs and colchicine on treating PPE in adults [9–12]

Study	Participants	Type of study	Drug used	Dosage	Duration	Inclusion criteria	Definition of success	Results
Horneffer et al. [9]	Adults > 18 years of age (*n* = 149)	RCT	Ibuprofen (*n* = 63)	600 mg every 6 h	10 days	Diagnosis of PPS [when ≥ 2 criteria met: (1) fever > 37.8 °C for > 8 h; (2) pericardial friction rub; and (3) considerable anterior chest pain without the presence of cardiac ischemia, differing from incisional pain] Procedures CABG, valve surgery, CABG in combination with valve surgery, and others	Primary outcome: Resolution of PPS symptoms, including assessing accumulation of PE with hemodynamic impact post-onset of symptoms	Significant differences between study groups and placebo group (*p *= 0.002)
			Indomethacin (*n* = 62)	25 mg every 6 h				
			Placebo (*n* = 24)	–				
Amoli et al. [11]	Adults ≥ 18 years of age (*n* = 149)	RCT	Colchicine, *n* = 74 (comparison with: placebo, *n* = 75)	1 mg/day	14 days	Mild (5 mm ≤ PE < 10 mm) or moderate (10 mm ≤ PE < 20 mm) PE as seen on transthoracic echocardiography (patients with minimal or severe PE excluded) Procedures CABG, valve replacement, aortoplasty, patent foramen ovale closure, septal myectomy, endarterectomy, and atrial appendage removal	Primary outcome: Decrease in mean PE volume and severity between 3^rd^ and 5^th^ week post-cardiac surgery	No significant differences between colchicine and placebo groups
Meurin et al. [10]	Adults ≥ 18 years of age (*n* = 262)	RCT	Diclofenac, *n* = 98 (comparison with: placebo, *n* = 98)	50 mg, twice a day	14 days	Moderate to large PE seen on first transthoracic echocardiography administered > 7 days postoperatively^a^ Procedures: CABG, valve replacement, valve repair, CABG and valve replacement/ repair combined, aortic aneurysm replacement, ascending aorta replacement, and other combinations of the above	Primary outcome: Decrease in severity of PE grade at end of 14 days of treatment. Other assessments: frequency of cardiac tamponade, of patients showing ≥ 1-grade decrease in PE severity, and average change in effusion width (mm)	No significant differences found between study group and placebo group
Meurin et al. [12]	Adults ≥ 18 years of age (*n* = 197)	RCT	Colchicine, *n* = 98 (comparison with: placebo, *n* = 99)	1 mg/day (loading dose of 1 mg twice a day for the first day given to patients weighing ≥ 70 kg)	14 days	Moderate to large PE seen on first transthoracic echocardiography administered > 7 days postoperatively^b^ Procedures CABG, valve replacement, valve repair, ascending aorta replacement, or combinations of the above	Primary outcome: mean decrease in severity of PE grade at the end of treatment, as seen on echocardiography. Other assessments: frequency of cardiac tamponade, of patients showing ≥ 1-grade decrease in PE severity, and mean change in width of the effusion (mm)	No significant differences found between study group and placebo group

Findings from Adult Literature

*RCT* randomized controlled trial, *CABG* coronary artery bypass graft, *PPS* postpericardiotomy syndrome, *PE* pericardial effusion

^a^The study defines this as a PE of grade ≥ 2 and “corresponding to a loculated effusion larger than 10 mm or a circumferential effusion of any size” [10]

^b^The study defines this as a PE of grade ≥ 2 and “corresponding to a loculated effusion larger than 10 mm or a circumferential effusion of any size” [12]

Meurin et al. [10] screened 5455 patients for PPE and of these, 196 were included (see Table 3 for inclusion criteria). These patients were randomly assigned to either a diclofenac or placebo group. Patients who had undergone CABG were additionally provided with “low-dose” aspirin. The intention-to-treat data analysis of the study findings revealed that while both groups showed a mean decrease in grade of PE severity, the difference in the magnitude of this change between the study groups (mean difference = − 0.28 grade) was not statistically significant (*p* = 0.11). Additionally, the number of patients who developed cardiac tamponade (*p* = 0.49) or showed a decrease of at least 1 grade in PE severity (*p* = 0.845) did not differ significantly between the two groups. Change in mean width of PE (in mm) was also found not to differ significantly between the two groups (*p* = 0.07) [10]. An overview of studies [9, 10] can be found in Table 3.

### Colchicine

Amoli et al. [11] assessed 154 patients who had undergone open-heart surgery, all of whom developed PPE and were thus enrolled in the study (see Table 3 for inclusion criteria). The patients were randomly assigned to either a colchicine or placebo group. Patients who had undergone CABG were additionally administered 80 mg of aspirin per day. The study did not find any significant differences between the two groups, either in terms of mean PE size or PE severity at the end of treatment (*p* = 0.844) or in terms of proportion of patients who showed at least a 1-grade reduction in PE severity as a result of treatment (*p* = 0.283) [11].

Meurin et al. [12] screened 8140 patients post-cardiac surgery for PE by means of a transthoracic echocardiography (TTE) and of these, 197 patients were included in the study (see Table 3 for inclusion criteria). Participants were randomly assigned to either a colchicine or placebo group. Patients who had undergone CABG were also regularly given “low-dose” aspirin. At the end of treatment, patients were given a second TTE. The intention-to-treat data analysis of the study findings revealed that mean change in PE grade from baseline did not differ significantly between the two groups (*p* = 0.23). Further, the number of patients who developed cardiac tamponade (*p* = 0.80) or showed a decrease of at least 1 grade in PE severity (*p* = 0.23) did not differ significantly between the two groups. Average change in width of PE (in mm) was also found not to differ significantly between the two groups (*p* = 0.27) [12]. An overview of studies [11, 12] can be found in Table 3.

## Discussion

PPE is an important and potentially life-threatening complication after pediatric cardiac surgery. In spite of this, the evidence in support of current drug treatment options for PPE is extremely limited and based almost entirely on the findings of small-scale RCTs like the study by Wilson et al. [8]. Moreover, the guidelines provided by relevant bodies like the European Society of Cardiology on how to treat PPE seem to be merely an expert opinion, based purely on experience and not on scientific evidence. In fact, even the references provided by these guidelines for the use of anti-inflammatory therapy or colchicine (in adjunct with aspirin or NSAIDs) are studies that are not of very high quality or describe the efficacy of the drug in prophylaxis as opposed to in the treatment of PPE [13].

To circumvent the problem of lack of pediatric literature, adult data were included with the intention of attempting to generalize the findings of such studies to the pediatric setting. However, there were several limitations to this approach. Much of the adult literature included in this review included samples of older adults (even though the age group of the cohorts in these studies is only specified to be above 18 years old, the procedures that the participants had undergone–CABG, for instance–are characteristic of an older population [14]). Previous studies have suggested that extremely young children (as opposed to the relatively older children on whom the RCT included in this review was conducted) and older adults (who seem to be the primary study population of the RCTs included in this review) have immune systems that do not function optimally, which makes these groups less prone to developing severe PPE, given that the immune system is often implicated in its etiology [3, 5]. This disparity in immune function between our population of interest and the population we have analyzed means that even though drugs like ibuprofen may achieve resolution of PPE in the latter, the same effect may not be observed in the former. This makes generalization of the findings from adult literature to the pediatric setting difficult and likely inadvisable.

Another issue with the adult literature is that the results are conflicting and possibly even biased by prophylactic NSAID administration (after CABG surgery) in a significant percentage of patients in both placebo and drug groups, as seen in the studies by Meurin et al. [10], Amoli et al. [11], and Meurin et al. [12]. The issue is further compounded by a possible risk of bias in reporting results found in the studies by Horneffer et al. [9] and Meurin et al. [12]. Study [9] has a composite endpoint and does not report its findings on individual parameters, making it difficult to ascertain if patients have benefited from the drug specifically in terms of PPE (one of the parameters). Study [12], on the other hand, specifies frequency of pericardial drainage after 30-day post-initiation of treatment as a secondary endpoint, but fails to report its findings for this endpoint. Moreover, the procedure in study [9] does not include administration of an echocardiography to the participants, with merely clinical signs as inclusion criteria, which further makes it difficult to draw any well-founded conclusions about the efficacy of the drug in question (ibuprofen and indomethacin) in the treatment of PPE.

An interesting finding did, however, result from an analysis of the adult literature. It is notable that in studies [10–12], aspirin, an NSAID, was administered to CABG patients in both groups, and these studies also did not find significant differences in treatment outcome between their study groups. On the other hand, part of the procedure in study [9] was to withhold any aspirin from participants and only provide non-aspirin analgesics on demand, and this study did in fact find significant differences in outcome between their study groups. This indicates that the etiology of PPE might be inflammatory; the administration of NSAIDs to participants in both placebo and drug groups may have reduced the apparent effect of the drug being studied in [10–12], since the anti-inflammatory effects of aspirin may have led to greater resolution of PPE in the placebo group than might otherwise have been observed. A major limitation to being able to draw this conclusion with greater certainty, however, is the aforementioned potential risk of bias found in [9]; the only study that did not administer any aspirin to its participants and also the only study to have found significant differences in treatment outcome. (It should be noted that the pediatric study by Wilson et al. [8] also withheld aspirin from its participants and also found significant results, but its sample size was too small (*n* = 21) for this finding to truly be of much significance, and as mentioned above, it also raised some concerns over risk of bias in randomization procedure and selection of reported results.)

The findings of this review were especially unexpected given the current prevalence in use of many of these drug treatments postoperatively, whether as treatment or prophylaxis for PPS. For instance, NSAIDs are commonly used for prevention of the development of PPS in children following cardiac surgery. A database search of PubMed in this case also served as a revelation; studies investigating the prophylactic use of NSAIDs (acetylsalicylic acid [15] and ibuprofen [16], both commonly employed in clinical practice) to prevent development of PPS in children found no significant results. That being said, the relatively low incidence of PPS as surmised from [2, 5] in the introduction section above may have a role to play in these findings. A low incidence of PPS means that even at a 100% efficacy of a drug, a large number of patients would need to be treated in order for PPS to be prevented in one patient—and since realistically, no drug is a 100% effective, the number of patients needed to be treated to show a significant effect of the drug would be higher still. Since such high numbers of patients needed to be treated are often difficult to achieve in practice, it might be worth not dismissing NSAIDs as a potential option for treatment or prophylaxis of PPS just yet. This can be supported by the possible confounding role NSAID administration may have played in the adult studies [10–12] included in this review; if administration of even “small amounts” of NSAIDs (as defined by the researchers of the above studies) was sufficient to skew the study results, then perhaps this can be used to a clinical advantage, especially given the relative safety of NSAID use even in children. Moreover, two additional studies retrieved during a PubMed database search found promising results using prophylactic NSAIDs (diclofenac) to prevent PPS development in adult populations [17, 18], so perhaps further research is needed to determine the true efficacy of NSAIDs as prophylaxis or treatment for PPS in the pediatric population.

**Fig. 3:**
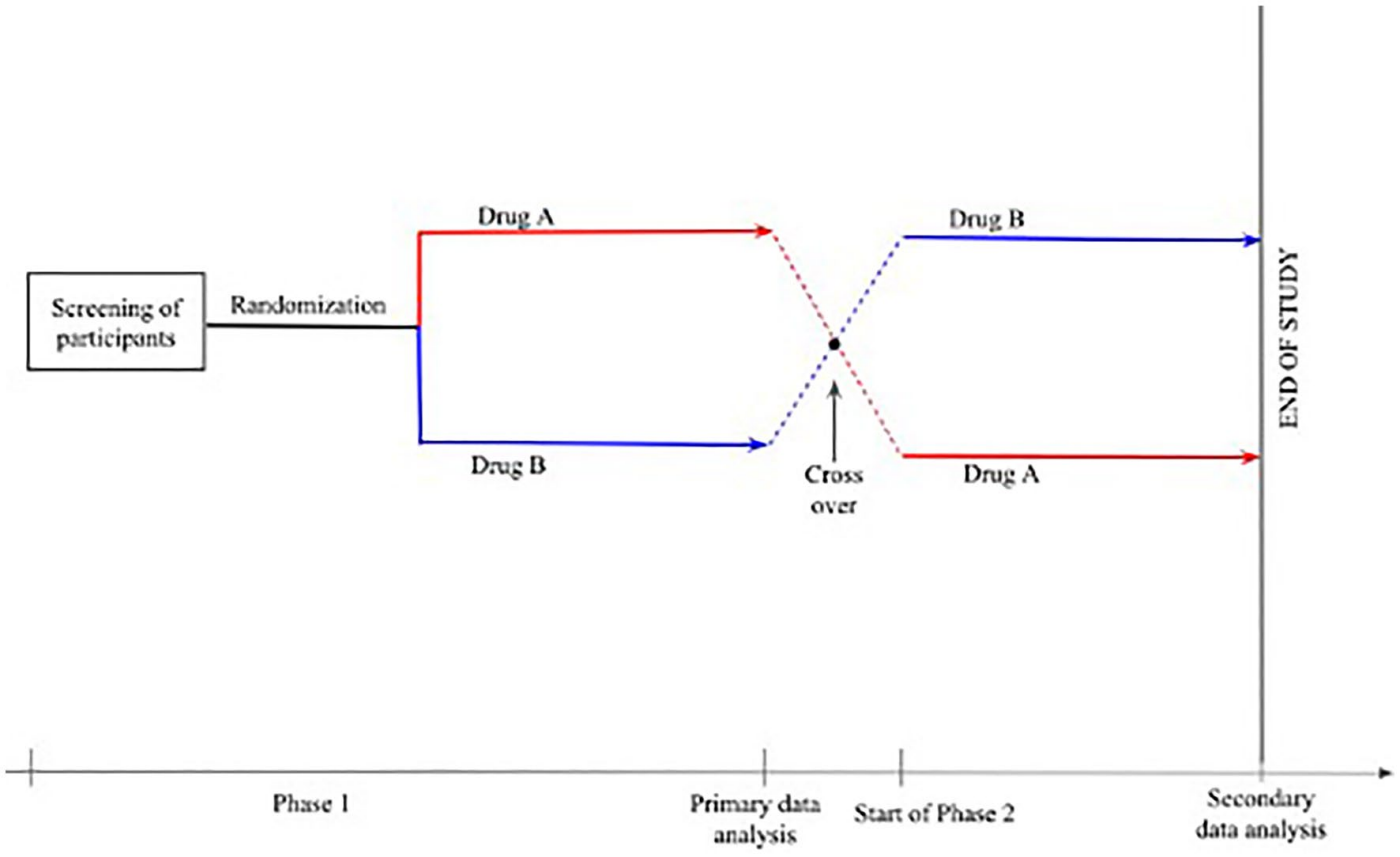
A hypothetical study design involving crossover trials to compare the efficacy of two drugs, A and B, in the treatment of PPE. Screening of participants would be followed by randomization into two study groups, one of which would receive drug A and the other, drug B. The end of Phase I of the study would be marked by primary data analysis and a crossover, wherein the two study groups would switch treatments. Phase 2 of the study would then commence and its end would be marked by the start of secondary data analysis. This would also mark the end of the study

Finally, this review may not have provided much concrete evidence for any of the three drugs investigated for the treatment of PPE, but it does shed light on the glaring lack of literature on the subject, indicating a need for future research. This is especially urgent for the pediatric setting, as children are not only more prone to developing PPE than the older individuals currently being studied [3, 5], but there is also rather scarce literature on treating PPE in children. There is thus a need for well-designed pediatric trials confirming the efficacy of prednisone, NSAIDs, and colchicine in treating PPE and evaluating the possible side effects of such treatments, which are currently being prescribed entirely on the basis of individual experiences with the drugs. Since placebo trials have already shown to be ineffective and even risky [9], crossover trials may be better suited for this purpose (see Fig. 3 for a hypothetical study design). It might also be useful to conduct a study investigating the incidence of PPS in two cohorts, one being administered NSAIDs prophylactically and the other, placebo. Researchers must, however, account for the high number of patients needed to be treated to prove drug efficacy in this case.

## Conclusion

The results of this study make apparent the fact that very little is currently known about what the best drug treatment for PPE might be and this is especially true for the pediatric setting. The inability to generalize the findings of adult literature to the pediatric setting further exacerbates the problem of the lack of pediatric evidence in support of any one drug treatment for PPE. Since PPE is a common postoperative complication with a possible impact on patient mortality, this severe lack of evidence must be rectified. There is, thus, an urgent need for good-quality clinical trials to investigate and compare the efficacy of corticosteroids, NSAIDs, and colchicine in treating pediatric PPE—a serious complication that modern medicine knows seriously little about.

## Appendix 1

Detailed search strategy

Search terms (PubMed)

1. “Pericardial Effusion/drug therapy” [Mesh]
2. “Pericardial Effusion/therapy” [Mesh]
3. “Postpericardiotomy Syndrome/drug therapy” [Mesh]
4. “Postpericardiotomy Syndrome/therapy” [Mesh]
5. “Pericardial Effusion” [Mesh]
6. “Postpericardiotomy Syndrome” [Mesh]
7. “postoperative pericardial effusion*”
8. “postoperative effusion*”
9. “pericardial effusion*”
10. “pericardiotomy syndrome”
11. ^#^1 OR #2 OR #3 … OR #10
12. “postoperative”
13. “postoperative complication*”
14. “postoperative care”
15. “cardiac surger*”
16. “cardiac surgical procedure*”
17. “heart surger*”
18. “Postoperative Complications/drug therapy” [Mesh]
19. “Postoperative Complications/therapy” [Mesh]
20. “Postoperative Care” [Mesh]
21. “Cardiac Surgical Procedures/adverse effects” [Mesh]
22. ^#^12 OR #13 OR #14 … OR #21
23. “Anti-Inflammatory Agents, Non-Steroidal” [Mesh]
24. “Ibuprofen” [Mesh]
25. “Adrenal Cortex Hormones” [Mesh]
26. “Colchicine” [Mesh]
27. “nonsteroidal anti-inflammatory agent*”
28. “nonsteroidal anti inflammatory agent*”
29. “NSAID”
30. “NSAIDs”
31. “nonsteroidal antiinflammatory agent*”
32. “non-steroidal anti-inflammatory agent*”
33. “aspirin-like agent*”
34. “aspirin like agent*”
35. “ibuprofen”
36. “adrenal cortex hormones”
37. “corticosteroid*”
38. “corticoid*”
39. “colchicine”
40. “Colchicine/therapeutic use” [Mesh]
41. “Colchicine/therapy” [Mesh]
42. “Anti-Inflammatory Agents, Non-Steroidal/therapeutic use” [Mesh]
43. “Ibuprofen/therapeutic use” [Mesh]
44. “Adrenal Cortex Hormones/drug therapy” [Mesh]
45. “Adrenal Cortex Hormones/therapeutic use” [Mesh]
46. “Adrenal Cortex Hormones/therapy” [Mesh]
47. ^#^23 OR #24 OR #25 … OR #46
48. ^#^11 AND #22 AND #47
49. English filter applied to #48
